# Hydrogen Attenuates Allergic Inflammation by Reversing Energy Metabolic Pathway Switch

**DOI:** 10.1038/s41598-020-58999-0

**Published:** 2020-02-06

**Authors:** Yinghao Niu, Qingrong Nie, Liping Dong, Jihua Zhang, Shu Fang Liu, Wei Song, Xiaopei Wang, Guangli Wu, Dongmei Song

**Affiliations:** 1 Departments of Otolaryngology and Clinical Biobank, the First Hospital of Hebei Medical University, Shijiazhuang, 050031 Hebei, China; 2 Department of Respiratory and Critical Care Medicine, Liangxiang Hospital of Beijing Fangshan District, Fangshan, 102401, Beijing, China; 3 Department of Psychiatry and Behavioural Neurosciences, Faculty of Health Sciences, McMaster University, ON L8N 3Z5 Hamilton, Canada

**Keywords:** Extracellular signalling molecules, Energy metabolism, Allergy, Asthma

## Abstract

Mechanisms mediating the protective effects of molecular hydrogen (H_2_) are not well understood. This study explored the possibility that H_2_ exerts its anti-inflammatory effect by modulating energy metabolic pathway switch. Activities of glycolytic and mitochondrial oxidative phosphorylation systems were assessed in asthmatic patients and in mouse model of allergic airway inflammation. The effects of hydrogen treatment on airway inflammation and on changes in activities of these two pathways were evaluated. Monocytes from asthmatic patients and lungs from ovalbumin-sensitized and challenged mice had increased lactate production and glycolytic enzyme activities (enhanced glycolysis), accompanied by decreased ATP production and mitochondrial respiratory chain complex I and III activities (suppressed mitochondrial oxidative phosphorylation), indicating an energy metabolic pathway switch. Treatment of ovalbumin-sensitized and challenged mice with hydrogen reversed the energy metabolic pathway switch, and mitigated airway inflammation. Hydrogen abrogated ovalbumin sensitization and challenge-induced upregulation of glycolytic enzymes and hypoxia-inducible factor-1α, and downregulation of mitochondrial respiratory chain complexes and peroxisome proliferator activated receptor-γ coactivator-1α. Hydrogen abrogated ovalbumin sensitization and challenge-induced sirtuins 1, 3, 5 and 6 downregulation. Our data demonstrates that allergic airway inflammation is associated with an energy metabolic pathway switch from oxidative phosphorylation to aerobic glycolysis. Hydrogen inhibits airway inflammation by reversing this switch. Hydrogen regulates energy metabolic reprogramming by acting at multiple levels in the energy metabolism regulation pathways.

## Introduction

In the past decade, the therapeutic potential of molecular hydrogen (H_2_) is increasingly recognized^1,2^. Studies have shown that H_2,_ administered by inhalation as hydrogen gas, by oral ingestion of hydrogen-rich water or by injection of hydrogen-rich saline (HRS), alleviates pathologies and improves outcomes in a wide range of pathological conditions^2–22^. However, the mechanisms mediating the protective effects of H_2_ are not well understood. H_2_ is small molecule that rapidly diffuses into cells and reacts with strong oxidants in cells^1^. The antioxidant activities could explain multiple beneficial effects of H_2_. H_2_ may act through disease-specific mechanisms and modulate specific pathways. Which mechanisms and pathways are modulated by H_2_ and how H_2_ may modulate these mechanisms and pathways remain essentially unknown.

Recently, the role of energy metabolic reprogramming in immune inflammatory response has drawn much attention^23–25^. Under normoxic conditions, cells produce ATP primarily through mitochondrial oxidative phosphorylation (OXPHOS). Under hypoxic conditions, cells produce ATP mainly through anaerobic glycolysis^25^. However, cancer cells produce ATP predominantly through glycolysis even in the presence of abundant oxygen (aerobic glycolysis)^26^. This energy metabolic switch from OXPHOS to aerobic glycolysis in cancer cells provides a means of quickly producing ATP and other metabolites that support the rapid growth of cancer cells. Activated immune inflammatory cells undergo much the same metabolic pathway switch^23–25^, which rapidly provides ATP and metabolic intermediates for the biosynthesis of immune and inflammatory proteins to lunch immune response. This metabolic reprogramming has been linked to multiple processes of immune activation^23–25,27^ and contributes significantly to many pathological processes in which inflammation plays a role. Modulation of this energy metabolic reprogramming could be a novel mechanism by which H_2_, and many other therapeutic agents, exert their anti-inflammatory and protective activities. However, neither the effect of H_2_ on energy metabolic reprogramming nor the mechanisms mediating the H_2_’s effects have been studied. Additionally, it remains unclear whether allergic inflammation is associated with an energy metabolic pathway switch.

Using asthma as a model of allergic inflammation, this study explored the possibility that H_2_ exerts its anti-inflammatory effect by modulating energy metabolic pathway switch, and studied the mechanisms by which H_2_ regulates energy metabolic reprogramming. We demonstrated that allergic airway inflammation is associated with a metabolic pathway switch from OXPHOS to aerobic glycolysis, and that H_2_ mitigates allergic airway inflammation by reversing this metabolic pathway switch. H_2_ modulates energy metabolic pathway switch by acting at multiple levels of the regulatory pathways that regulate energy metabolism. Our studies reveal a new mechanism by which H_2_ exerts its anti-inflammatory activity and protects against allergic inflammation.

## Results

### Energy metabolic pathway switch in asthmatic patients

Prior studies have shown that asthmatic patients had elevated serum and sputum levels of lactate and that cell from asthmatic patients produced higher amounts of lactate upon stimulation^28,29^. OVA sensitization and challenge upregulated glycolysis enzyme expression in rat lungs^30^. However, whether an enhanced glycolysis is accompanied by a reduced OXPHOS activity (whether an energy metabolic pathway switch occurs) remains unclear. To study whether H_2_ may regulate energy metabolic reprogramming, we first ascertained whether allergic airway inflammation is associated with an energy metabolic reprogramming.

**Figure 1:**
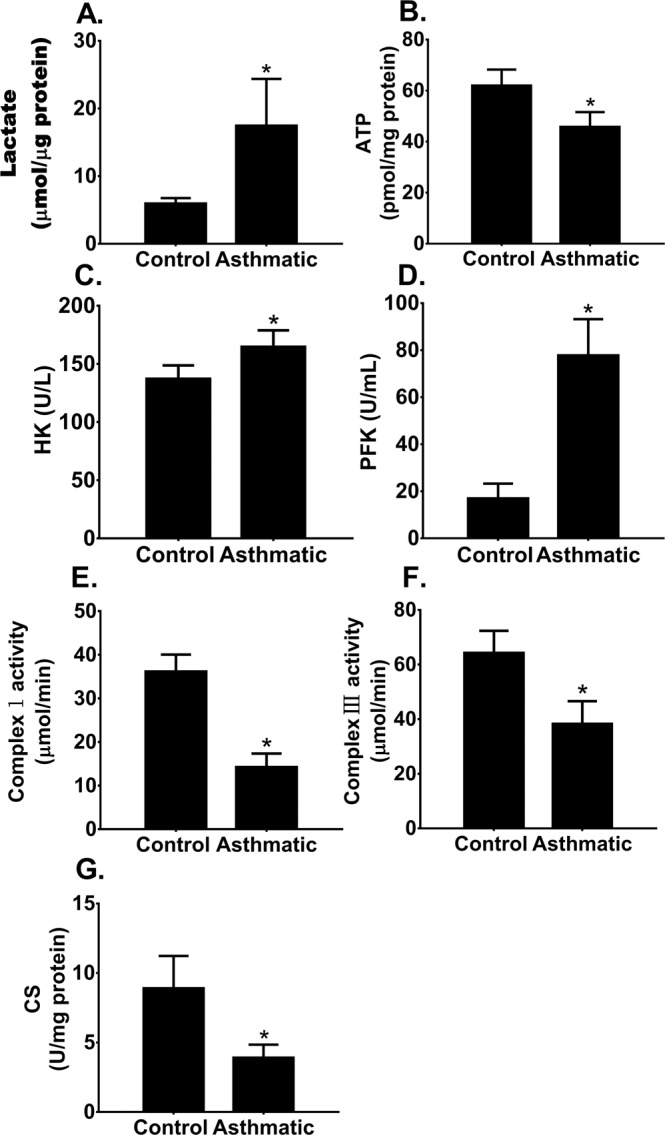
Energy metabolic pathway switch in patients with asthma. Peripheral blood mononuclear cells (PBMCs) were isolated from control and asthmatic subjects. PBMC glycolysis and mitochondrial oxidative phosphorylation (OXPHOS) activities were characterized and compared. Means ± SEM of 7 subjects per group. *p < 0.05, compared to control subjects (unpaired t test). (**A**,**B**). PBMC lactate and ATP production. (**C**,**D**). PBMC hexokinase (HK) and phosphofructokinase (PFK) activities. (**E**,**F**) PBMC mitochondrial OXPHOS complex I and complex III activities. (**G**) PBMC citrate synthase (CS) activity.

We characterized energy metabolic pathways in PBMCs of asthmatic and control subjects. Table 1 summarizes the demographic information for the two groups. There were no differences in age, sex, BMI, or smoking history between the 2 groups. Patients with asthma displayed an increased blood eosinophil count, and lower FEV1 and FEV1/FVC ratio. PBMCs from asthmatic patients showed a significantly increased lactate production (Fig. 1A) and decreased ATP production (Fig. 1B). Consistent with the increased lactate and decreased ATP production, asthmatic PBMCs had significantly increased HK and PFK activities (Fig. 1C,D), two key enzymes that catalyze the glycolysis reaction, significantly decreased mitochondrial OXPHOS complex I and III activities (Fig. 1E,F), which catalyze the oxidation-reduction in the mitochondrial electron transport chain, and a decreased CS activity (Fig. 1G), the pace-making enzyme in the first step of the Krebs cycle. The enhanced glycolytic activity and repressed OXPHOS activity in association with an increased lactate and decreased ATP production in asthmatic PBMCs suggest that asthma is associated with an energy metabolic pathway switch from OXPHOS to aerobic glycolysis.

**Table 1:** Clinical Characteristics of Participants.

	Healthy subjects	Asthmatics	*P value*
Number	7	7	
Age (years)	52.43 ± 4.35	57.57 ± 8.41	0.1765
Male:Female	2:5	3:4	—
BMI (kg/m^2^)	28.10 ± 2.32	25.28 ± 4.20	0.1459
Ex-smoker, no. (%)	2 (29)	2 (29)	—
Blood eosinophils ( × 10^9^)	0.17 ± 0.11	0.32 ± 0.09*	0.0162
FEV1 (L)	2.30 ± 0.28	2.16 ± 0.76	0.6556
FEV1 (% predicted)	83.24 ± 10.04	66.24 ± 11.14*	0.0111
FVC (L)	2.77 ± 0.45	3.27 ± 1.10	0.2875
FVC (% predicted)	83.63 ± 10.13	89.43 ± 15.22	0.4177
FEV1/FVC ratio(%)	83.54 ± 6.20	59.69 ± 5.23*	<0.0001

BMI, body mass index; Ex-smoker was defined as not smoking for at least six months.

*p < 0.05 compared to control subjects.

### Energy metabolic pathway switch in mouse model of allergic airway inflammation

**Figure 2:**
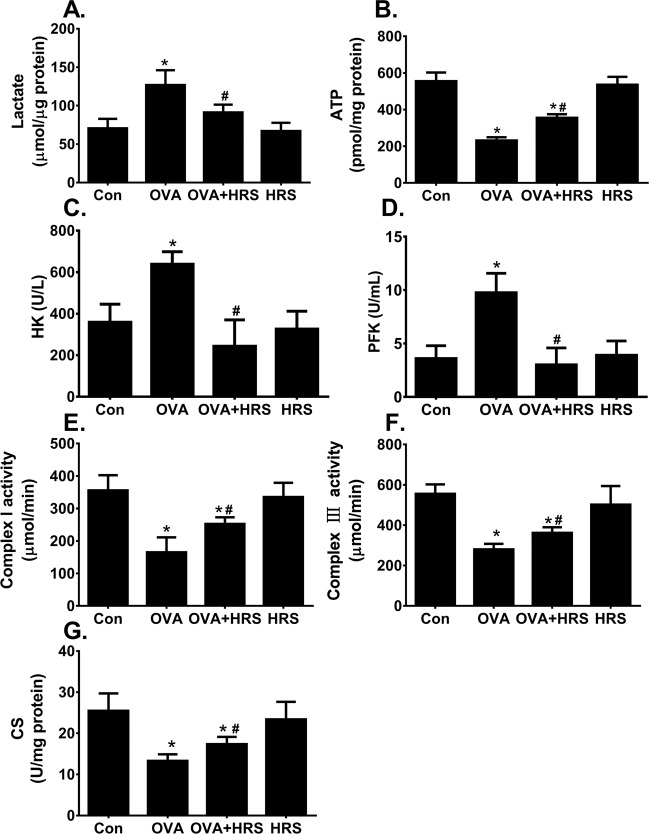
H_2_ reverses OVA sensitization and challenge-induced energy metabolic pathway switch. Mice in control (Con) and hydrogen-rich saline (HRS) groups were sham sensitized and challenged. Mice in ovalbumin (OVA) and OVA + HRS groups were sensitized and challenged with OVA. Mice in HRS or HRS + OVA group was administered HRS (6 ml/ kg /day, i.p.). At 24 hours after final OVA challenge, lungs were collected. Lung tissue levels of lactate, ATP, HK, PFK and CS activities, and lung mitochondrial OXPHOS complex I and III enzyme activities were measured. Means ± SEM of 5 mice per group. *p < 0.05, compared to control group. ^#^p < 0.05, compared to OVA group (one-way ANOVA). **(A**,**B**) Lung lactate and ATP production. (**C**,**D**) Lung HK and PFK activities. (**E**,**F**) Lung mitochondrial OXPHOS complex I and III activities. (**G**) Lung CS activity.

We used a well-established mouse model of OVA sensitization and challenge-induced airway inflammation to study the mechanisms by which H_2_ modulates allergic inflammation-associated energy metabolic pathway switch. OVA-sensitized and challenged mice developed significant airway inflammation, and showed a significantly increased lung tissue level of lactate, and decreased lung issue level of ATP production (Fig. 2A,B). The increased lactate production was accompanied by increased HK and PFK activities (Fig. 2C,D), whereas the decreased ATP production was accompanied by decreased mitochondrial complex I and III activities (Fig. 2E,F), and a decreased CS activity (Fig. 2G). Thus, allergic airway inflammation is associated with an energy metabolic pathway switch from OXPHOS to aerobic glycolysis in mouse model.

### H_2_ reverses allergic inflammation-associated energy metabolic pathway switch

To examine whether H_2_ modulates allergic inflammation-associated energy metabolic pathway switch, we treated mice with HRS for 7 days. HRS had no effects on OXPHOS and glycolytic activities in control mice (Fig. 2A,B, HRS), but prevented the elevation in lactate and reduction in ATP production in lungs of OVA-sensitized and challenged mice (Fig. 2A,B, OVA vs HRS + OVA). Consistently, HRS attenuated the increase in HK and PFK activities (Fig. 2C,D, OVA vs HRS + OVA), the decrease in mitochondrial OXPHOS complex I and III activities (Fig. 2E,F, OVA vs HRS + OVA), and the decrease in CS activity (Fig. 2G, OVA vs HRS + OVA) in lungs of OVA-sensitized and challenged mice. These results suggest that H_2_ reverses OVA sensitization and challenge-induced metabolic pathway switch by inhibiting the stimulation of glycolytic enzyme activities and by relieving the inhibition of OXPHOS enzyme activities.

### H_2_ counter-regulates OVA sensitization and challenge-induced HIF-1α upregulation and PGC-1α downregulation

**Figure 3:**
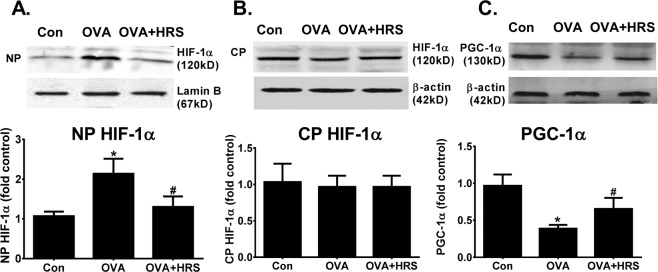
H_2_ counter-regulates OVA sensitization and challenge-induced HIF-1α upregulation and PGC-1α downregulation. Mice in Con, OVA or OVA + HRS group were sensitized and challenged, and treated with HRS as described above. Lung tissue levels of hypoxia inducible factor (HIF)-1α and peroxisome proliferator-activated receptor-γ coactivator (PGC)-1α were analyzed. The Western blot HIF-1α or PGC-1α bands (upper panel) were quantified using densitometry and expressed as fold change over control group (bar graphs). Means ± SEM of 5 mice per group. *p < 0.05, compared to control group. ^#^p < 0.05, compared to OVA group (one-way ANOVA). (**A**) Nuclear HIF-1α protein content. (**B**) Cytoplasmic HIF-1α protein content. (**C**) PGC-1α protein expression. NP, nuclear protein. CP, cytoplasmic protein. Cropped blots are displayed. The original blots are shown in Supplementary Fig. 1A–C.

Genes encoding glycolytic or OXPHOS enzymes are regulated by HIF-1α or PGC-1α^31–33^. We examined the effects of HRS, and OVA sensitization and challenge on HIF-1α and PGC-1α expressions and activities. OVA sensitization and challenge increased HIF-1α nuclear translocation (stimulated HIF-1α activity), which was inhibited by HRS treatment (Fig. 3A,B). By contrast, OVA sensitization and challenge downregulated PGC-1α protein expression, and HRS treatment reversed this downregulation (Fig. 3C). Considering that HIF-1α regulates the expression of numerous genes encoding enzymes that make up the glycolytic machinery^31,32^ and that PGC-1α regulates the expression of numerous genes mediating mitochondrial biogenesis and OXPHOS^33^, these results suggest that OVA sensitization and challenge may cause energy metabolic pathway switch by upregulating HIF-1α and downregulating PGC-1α. H_2_ may reverse this pathway switch by counter-regulating this up- and down-regulation.

### H_2_ abrogates sirtuin downregulation in OVA-sensitized and challenged mice

**Figure 4:**
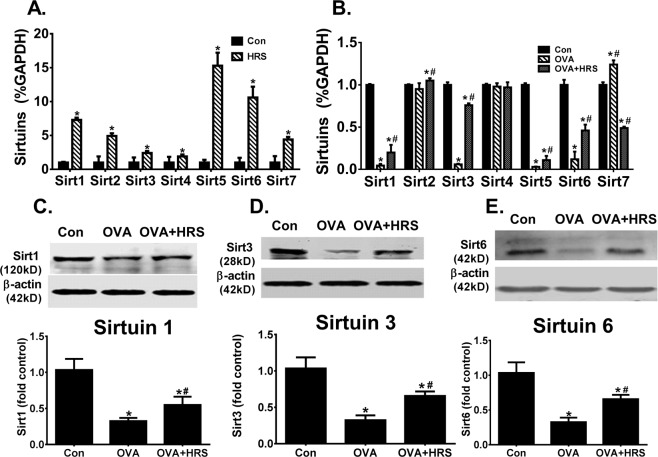
H_2_ regulates sirtuin expression. Mice in Con, HRS, OVA or OVA + HRS group were sensitized and challenged, and treated with HRS as described above. Lung tissue levels of sirtuins (Sirt) 1, 2, 3, 4, 5, 6 and 7 mRNA expression were quantified by qRT-PCR and expressed as sirtuin/GAPDH (glyceraldehyde 3-phosphate dehydrogenase) ratio, or lungs tissue levels of sirtuins 1, 3 and 6 proteins were quantified by Western blots. Means ± SEM of 5 mice per group. *p < 0.05, compared to control group. ^#^p < 0.05, compared to OVA group (unpaired t test or one-way ANOVA). (**A**) Sirtuin mRNA expression in control lungs. (**B**) Sirtuin mRNA expression in OVA-sensitized and challenged lungs. (**C–E**) Sirtuins 1, 3 and 6 protein expression in OVA-sensitized and challenged lungs. The Western blot bands (upper panel) were quantified using densitometry and expressed as fold change over control group (bar graphs). Cropped blots are displayed. The original blots are shown in Supplementary Fig. 2A–C.

**Figure 5:**
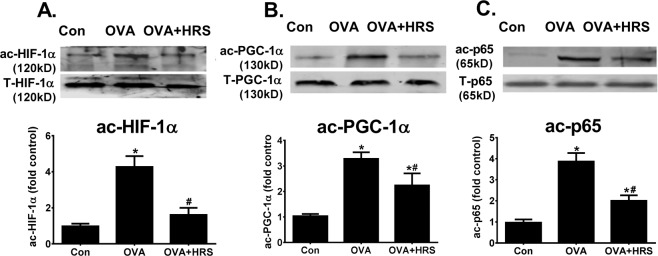
H_2_ inhibits OVA sensitization and challenge-induced HIF-1α, PGC-1α and p65 acetylation. Mice in Con, OVA or OVA + HRS group were sensitized and challenged, and treated with HRS as described above. Lung tissue levels of total (T-HIF-1α, T-PGC-1α and T-p65) and acetylated (ac-HIF-1α, ac-PGC-1α and ac-p65) HIF-1α, PGC-1α and NF-κB p65 were quantified by Western blots. Means ± SEM of 5 mice per group. *p < 0.05, compared to control group. ^#^p < 0.05, compared to OVA group (one-way ANOVA). (**A–C**) Lung tissue levels of total and acetylated HIF-1α, PGC-1α and p65 protein. The ac-HIF-1α, ac-PGC-1α and ac-p65 bands were quantified using densitometry and expressed as fold change over control group. Cropped blots are displayed. The original blots are shown in Supplementary Fig. 3A–C.

The sirtuin family of proteins is an upstream modulator of HIF-1α and PGC-1α^34–36^. We assessed the effects of H_2_ on sirtuin expression. HRS upregulated basal mRNA expression of all 7 mammalian sirtuins in control lungs (Fig. 4A). OVA sensitization and challenge variably regulated sirtuin mRNA expression, downregulating sirtuins 1, 3, 5 and 6 markedly, upregulating sirtuin 7 moderately, and causing little changes in sirtuins 2 and 4 expression (Fig. 4B). At protein level, OVA sensitization and challenge downregulated sirtuin 1, 3 and 6 expressions in the lungs (Fig. 4C–E). HRS treatment partially reversed sirtuin 1, 3, 5 and 6 mRNA downregulation (Fig. 4B), inhibited sirtuin 7 mRNA upregulation (Fig. 4B), and reversed sirtuins 1, 3 and 6 protein downregulation (Fig. 4C–E). In parallel to sirtuin downregulation, levels of acetylated HIF-1α, acetylated PGC-1α and acetylated p65, major targets of sirtuin deacetylase, were significantly increased in the same lungs of OVA-sensitized and challenged mice (Fig. 5A–C), indicating a reduced sirtuin deacetylase activity. Treatment of OVA-sensitized and challenged mice with HRS reduced lung levels of acetylated HIF-1α, PGC-1α and p65, indicating restoration of sirtuin deacetylase activity (Fig. 5A–C).

### H_2_ restores mitochondrial NAD production in OVA-sensitized and challenged mice

**Figure 6:**
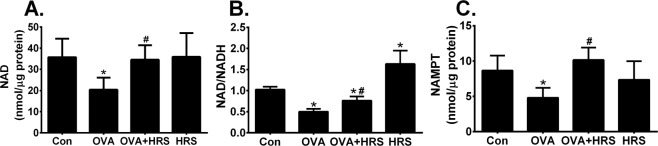
H_2_ relieves OVA sensitization and challenge-induced suppression of NAD production. Mice in Con, HRS, OVA or OVA + HRS group were sensitized and challenged, or/and treated with HRS as described above. Lung mitochondrial levels of nicotinamide adenine dinucleotide (NAD), NAD/NADH ratio and nicotinamide phosphoribosyltransferase (NAMPT) content were measured. Means ± SEM of 5 mice per group. *p < 0.05, compared to control group. ^#^p < 0.05, compared to OVA group (one-way ANOVA). (**A**) Lung mitochondrial NAD production. (**B**) Lung mitochondrial NAD/NADH ratio. (**C**) Lung mitochondrial NAMPT expression.

H_2_ and OVA sensitization and challenge may regulate sirtuin deacetylase activity by altering its substrate availability. NAD is an obligatory substrate for sirtuin deacetylase activity, and NAMPT is the rate-limiting enzyme in NAD biosynthesis^36,37^. We measured lung mitochondrial levels of NAD, NAD/NADH and NAMPT. OVA sensitization and challenge significantly reduced lung mitochondrial levels of NAD, NAD/NADH and NAMPT, which were prevented by HRS treatment (Fig. 6A–C, OVA vs OVA + HRS), suggesting that H_2_ relieves OVA sensitization and challenge-induced repression of sirtuin deacetylase activity by improving NAD synthesis or/and availability.

### H_2_ ameliorates allergic airway inflammation

**Figure 7:**
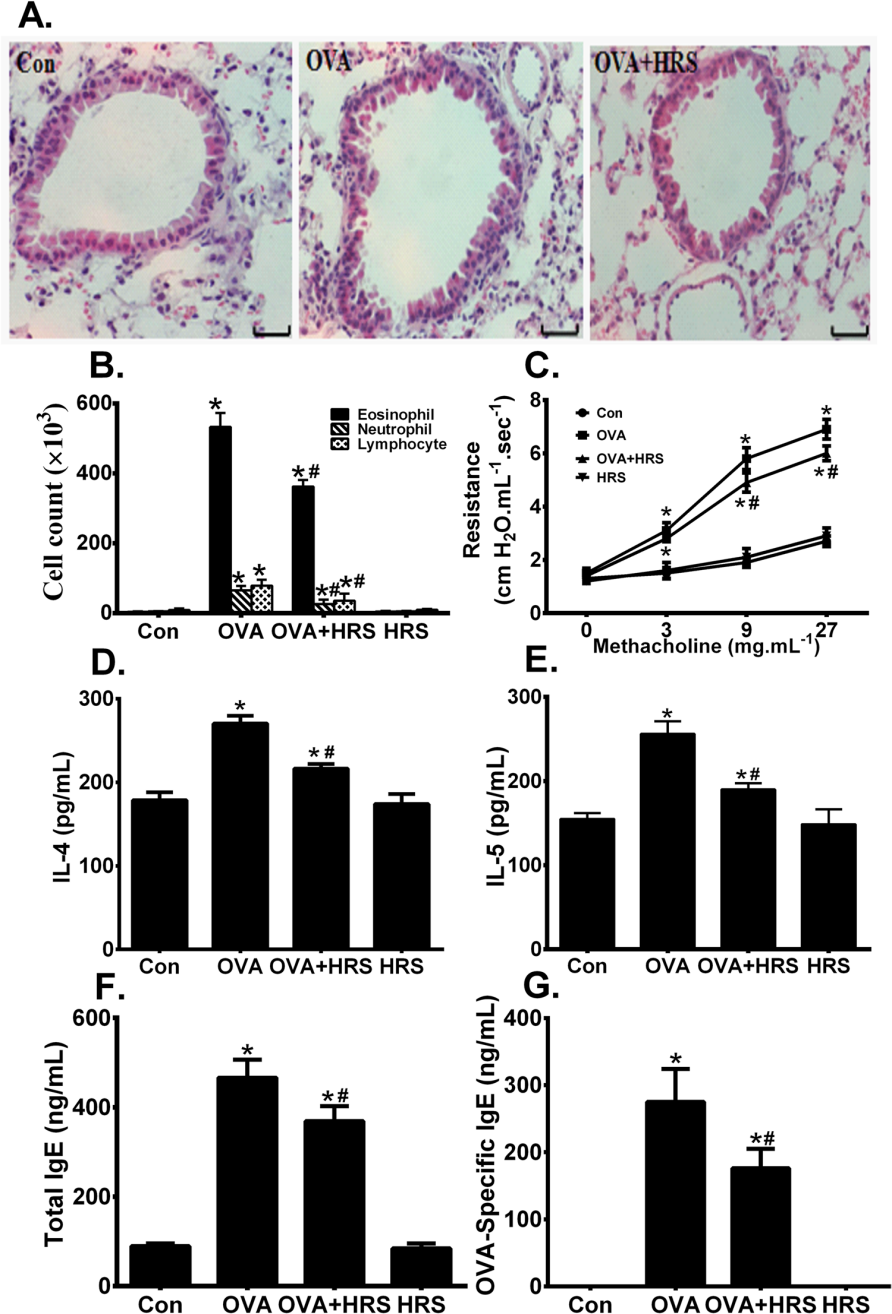
H_2_ ameliorates allergic airway inflammation. Mice in Con, HRS, OVA or OVA + HRS group were sensitized and challenged, or/and treated with HRS as described above. Lung and airway histology was evaluated, airway responsiveness assessed, bronchoalveolar lavage fluid (BALF) inflammatory cells counted, and BALF IgE, IL-4 and IL-5 measured. Means ± SEM of 5 mice per group. *p < 0.05, compared to control group. ^#^p < 0.05, compared to OVA group (one-way ANOVA). **(A**) Lung and airway histology. Scale bar = 20 μm. (**B**) BALF inflammatory cell count. (**C**) Airway responsiveness. (**D**,**E**) BALF levels of IL-4 and IL-5. (**F**,**G**) BALF total and OVA-specific IgE.

**Figure 8:**
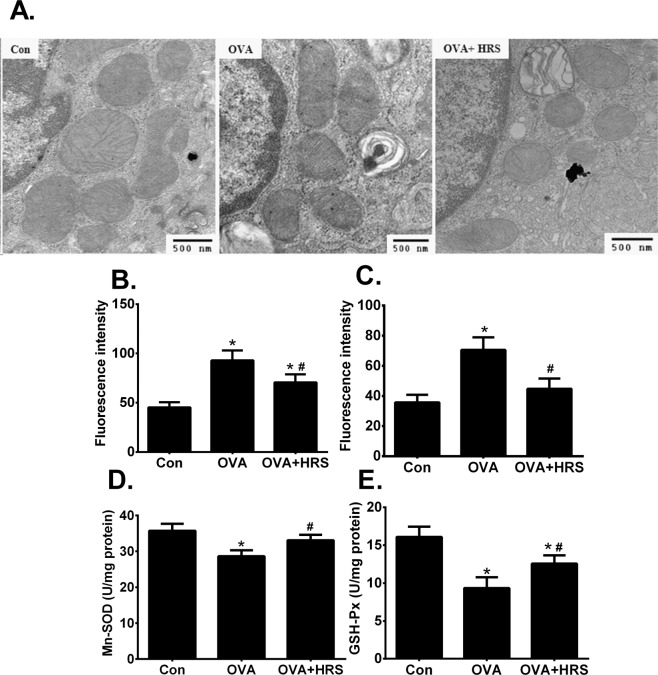
H_2_ rebalances the imbalance between oxidant and antioxidant. Mice in Con, OVA or OVA + HRS group were sensitized and challenged, and treated with HRS as described above. Mitochondrial morphology was assessed by transmission electron microscopy. BALF leukocyte and lung mitochondrial ROS production was measured using fluorescent probe and expressed as fluorescence intensity. Lung mitochondrial Mn-superoxide dismutase (Mn-SOD) and glutathione peroxidase (GSH-Px) activities were determined. Means ± SEM of 5 mice per group. *p < 0.05, compared to control group. ^#^p < 0.05, compared to OVA group (one-way ANOVA). (**A**) Lung mitochondrial morphology. Magnification, x 30000. (**B**,**C**) BALF leukocyte and lung mitochondrial ROS production. (**D**,**E**) Lung mitochondrial Mn-SOD and GSH-Px activities.

Reversal of metabolic pathway switch by HRS treatment was associated with an improved pathology and functional outcome. OVA sensitization and challenge caused significant airway inflammation, as indicated by airway inflammatory cell infiltration, and epithelial injury and detachment (Fig. 7A), two major characteristics of the structural changes in allergic airway inflammation. OVA-sensitized and challenged mice had significantly increased number of BALF inflammatory cell count, particularly eosinophil count (Fig. 7B), airway hyperresponsiveness (Fig. 7C), significantly elevated BALF levels of IL-4 and IL-5 (Fig. 7D,E), and elevated BALF total and OVA-specific IgE (Fig. 7F,G). Treatment of OVA-sensitized and challenged mice with HRS ameliorated the pathological changes in airways (Fig. 7A), reduced the number of BALF inflammatory cell count (Fig. 7B), alleviated airway hyperresponsiveness (Fig. 7C), and reduced BALF levels of cytokines and IgE (Fig. 7D–G). HRS also alleviated oxidant stress in the lungs. HRS treatment mitigated changes in mitochondrial morphology (Fig. 8A), inhibited the increases in BALF leukocyte and lung mitochondrial ROS production (Fig. 8B,C), and attenuated the decreases in mitochondrial Mn-SOD and GSH-Px activities (Fig. 8D,E) in OVA-sensitized and challenged mice. Thus, reversal of metabolic pathway switch by HRS treatment is associated with an improvement in airway inflammation and airway hyperresponsiveness.

## Discussion

This study demonstrates that allergic airway inflammation is associated with an energy metabolic pathway switch, and that H_2_ mitigates allergic airway inflammation by reversing this energy metabolic pathway switch. We showed that PBMCs from patients with asthma and lungs from mice with OVA sensitization and challenge-induced airway inflammation displayed an increased level of lactate and decreased level of ATP productions, indicating an enhanced glycolysis and repressed OXPHOS. The increased lactate production was accompanied by increased glycolytic enzyme, PFK and HK, activities, whereas the decreased ATP production was accompanied by decreased mitochondrial OXPHOS complex I and III activities, and a decreased CS activity. Thus, we have demonstrated that allergic airway inflammation is associated with an energy metabolic pathway switch from OXPHOS to aerobic glycolysis. The biochemical basis for this switch is likely to be an upregulation of glycolytic enzyme activities and a downregulation of OXPHOS enzyme activities.

Treatment of OVA-sensitized and challenged mice with HRS reversed the increase in lactate production and decrease in ATP production. HRS treatment also reversed the increase in PFK and HK activities and the decrease in mitochondrial OXPHOS complex I and III, and CS activities. Concomitantly, HRS treatment mitigated allergic airway inflammation and airway hyperreponsiveness in these mice. This result suggests that H_2_ inhibits airway inflammation by reversing the energy metabolic pathway switch. H_2_ reverses energy metabolic switch by inhibiting glycolytic enzyme activities and by stimulating mitochondrial OXPHOS enzyme activities. Our data demonstrates a novel mechanism that H_2_ exerts its anti-inflammatory action by regulating energy metabolic reprogramming.

H_2_ may modulate the allergic inflammation-associated energy metabolic reprogramming by multiple mechanisms. OVA sensitization and challenge stimulated PFK and HK activities, and suppressed mitochondrial OXPHOS complex I and III activities. HRS treatment inhibited this stimulation and relieved this suppression, indicating that H_2_ may directly modulate glycolytic and OXPHOS activities. H_2_ may also act at upstream regulatory elements. OVA sensitization and challenge upregulated HIF-1α activity and downregulated PGC-1α expression. HRS treatment prevented the up- and down-regulation. It is well documented that HIF-1α mediates the transcription of multiple genes in the glycolytic pathway, including PFK and HK genes^35,36^. PGC-1α regulates the expression of multiple genes that mediate OXPHOS and mitochondrial biogenesis^33,35–37^. OVA sensitization and challenge may cause metabolic pathway switch by HIF-1α-mediated upregulation of HK and PFK, as well as other glycolytic enzymes, and by PGC-1α-mediated downregulation of OXPHOS enzymes. H_2_ may reverse the energy metabolic pathway switch by counter-regulating the up- and down-regulation.

The sirtuin family of proteins is upstream regulator of HIF-1α and PGC-1α^34–37^, and may play important roles in H_2_-mediated modulation of energy metabolic switch and in H_2_-mediated protection against allergic airway inflammation. OVA sensitization and challenge downregulated sirtuin expression and activity, and concomitantly caused energy metabolic pathway switch and airway inflammation. HRS treatment prevented sirtuin downregulation and restored sirtuin deacetylase activity, which were associated with a reversal of energy metabolic switch and an improvement in airway inflammation in OVA-sensitized and challenged mice. Sirtuin deacetylase activity is regulated by an obligatory cofactor, NAD. NAD is synthesized by the NAD salvage pathway, in which NAMPT is a rate-limiting enzyme^36,37^. Consistent with a downregulated sirtuin deacetylase activity, tissue levels of NAD and NAMPT were significantly lower in OVA-sensitized and challenged mice, but were restored to control levels in HRS-treated, OVA-sensitized and challenged mice. HRS treatment also upregulated basal expression of all 7 mammalian sirtuins, and increased basal NAD level in the lungs.

It is well established that sirtuins regulate metabolism through deacetylation of target proteins^33–39^. Both HIF-1α and PGC-1α are targets of sirtuin 1^34–38^. Deacetylation of HIF-1α by sirtuin 1 represses HIF-1α activity, whereas deacetylation of PGC-1α by sirtuin 1 increases its transcriptional activity^34,35^. Sirtuin 6 has similar activities^38,39^. It is possible that OVA sensitization and challenge suppresses sirtuin activity, which increases HIF-1α acetylation and activity, resulting in an upregulated glycolytic enzymes and an enhanced glycolysis. Concurrently, suppression of sirtuin activity increases PGC-1α acetylation and inhibits PGC-1α transcriptional activity, resulting in a reduced OXPHOS. The two mechanisms work in parallel, leading to energy metabolic pathway switch. HRS treatment restores sirtuin expression and activity, decreases HIF-1α transcriptional activity, increases PGC-1α transcriptional activity, inhibits glycolysis and stimulates OXPHOS, reversing energy metabolic pathway switch and airway inflammation in OVA-sensitized and challenged mice. Thus, H_2_ may regulate the energy metabolic pathway switch by acting at multiple levels in the regulatory pathways of energy metabolism.

Prior studies have reported that asthmatic patients had elevated serum and sputum levels of lactate and that cell from asthmatic patients produced higher amounts of lactate upon stimulation^28,29^, suggesting an enhanced glycolysis. However, these prior studies did not address the question whether the enhanced glycolysis is accompanied by a repressed OXPHOS activity (whether an energy metabolic pathway switch occurs). Additionally, the biochemical and molecular mechanisms underlying the metabolic reprogramming in context of allergic airway inflammation remain largely unclear. Our study extends these previous studies by demonstrating a link between allergic airway inflammation and energy metabolic pathway switch. More importantly, we elucidated the mechanisms linking allergic airway inflammation and energy metabolic pathway switch. Furthermore, we demonstrated a novel mechanism that H_2_ alleviates allergic airway inflammation by reversing the energy metabolic switch.

## Conclusion

Allergic airway inflammation is associated with an energy metabolic pathway switch from mitochondrial oxidative phosphorylation to aerobic glycolysis. H_2_ reverses this metabolic pathway switch and mitigates allergic airway inflammation. H_2_ appears to regulate the allergic inflammation-associated energy metabolic pathway switch by multiple mechanisms. H_2_ directly inhibits glycolytic enzyme activities and stimulates mitochondrial OXPHOS enzyme activities. H_2_ acts at upstream regulatory elements and regulates co-factor production in the energy metabolism regulation pathways, reversing the upregulation of glycolytic enzyme activities and the downregulation of OXPHOS enzyme activities, and energy metabolic pathway switch. Our data uncovers a novel mechanism that H_2_ mitigates allergic airway inflammation by reversing energy metabolic pathway switch.

## Methods

### Human studies

Seven control and 7 asthmatic subjects were enrolled into the study based on Global Initiative for Asthma (GINA) criteria^40^. The study protocol was approved by the Ethics Review Board of Liangxiang Hospital of Beijing Fangshan District, and carried out in accordance with the declaration of Helsinki. A written consent was obtained from each participant. Asthmatic patients had typical symptoms and physiological evidence of asthma, and a significant airway hyperresponsiveness (a provocative dose of inhaled methacholine causing a 20% reduction in FEV1 of <8 mmol) or a positive response to a bronchodilator (FEV1 improvement of ≥12% and ≥200 ml). Asthmatic patients had not been treated with corticosteroids in the last 3 months. Participants were excluded if they had pulmonary disease other than asthma, inflammation in other organs, smoked within the prior six months, any comorbidity and medication that could potentially alter systemic inflammation, or other significant diseases that in the opinion of the investigators would interfere with study participation. Control participants had no diagnosis of asthma, had no history of respiratory diseases and were not atopic.

Heparinized peripheral blood was collected from each participant following informed consent. Peripheral blood mononuclear cells (PBMCs) were isolated by Ficoll-Paque Plus (GE Healthcare Life Sciences, Pittsburgh, PA, USA) density-gradient centrifugation, as we have previously described^41^.

### Animal studies

An established mouse model of allergic airway inflammation was used^42^. Study protocols were approved by Institutional Animal Care and Use Committee of Hebei Medical University, and complied with regulations of the Ministry of Health of China and the US National Institute of Health guidelines. FVB mice (8–10 weeks) were obtained from Vital River Laboratory Animal Technology Co. Ltd (Beijing, China), and randomly divided into control, hydrogen-rich saline (HRS), ovalbumin (OVA) and OVA + HRS groups. Mice in control group and HRS group were sham-sensitized by injection with phosphate buffered saline (PBS) and sham-challenged by PBS inhalation. Mice in the other 2 groups were sensitized by intraperitoneal injection of 25 μg per mouse OVA emulsified in 2.5 mg aluminum hydroxide in 250 μl PBS on days 1, 9 and 14, and then challenged by aerosolized 1% OVA in PBS for 20 minutes per day on days 21 to 27. Mice in OVA + HRS and HRS groups were administered HRS (6 ml/kg/day, i.p.) on days 21 to 27. On day 28, bronchoalveolar lavage (BAL) was performed by slowly delivering 0.8 ml of pre-warmed (~37 °C) PBS through a polyethylene tube inserted into trachea followed by gentle suction. This procedure was repeated 3 times on each mouse, and the collected PBS was pooled. Lungs and BAL fluid (BALF) were collected for further analyses.

### Hydrogen-rich saline (HRS) production

HRS was prepared by dipping a plastic-shelled stick consisting of metallic magnesium (99.9% pure) and natural stones (Doctor SUISOSUI, Friendear Inc., Tokyo, Japan) into sterilized saline. Fifty μl of HRS was injected into a lid-sealed glass container. H_2_ level in the gas phase (1 ml) was determined using a biogas analyzer with a semiconductor sensor (TRI lyzer mBA-3000; Taiyo KK, Osaka, Japan). H_2_ concentration was expressed as parts per million (ppm). HRS was freshly prepared every other day. H_2_ concentration in HRS was maintained between 0.3 and 0.4 ppm during the experiment.

### Determination of cellular and tissue levels of lactate and ATP production, and of energy metabolic enzyme activities

Mouse lung tissues (100 mg) and human PBMCs (1 × 10^6^ cells) were homogenized in phosphate buffer (pH 7.4) using a DIAX 600 tissue homogenizer (Heidolph, Schwabach, Germany) on ice. The homogenates were centrifuged at 2500 rpms at 4 °C for 10 minutes. The supernatants were collected and protein concentration determined by BCA method. Mouse lung and human PBMC levels of lactate and ATP productions were measured using lactate and ATP assay kits (Nanjing Jiancheng Bio-engineering Institute, Nanjing, China) following manufacturer’s protocols.

Lung and PBMC levels of hexokinase (HK), phosphofructokinase (PKF) and citrate synthase (CS) activities were measured using corresponding assay kit (Nanjing Jiancheng Bio-engineering Institute, Nanjing, China). To measure HK activity, 30 μl of each sample was mixed with and incubated in 1000 μl reaction Mix containing HK substrate and coenzymes at 37 °C. Absorbance at 340 nm (A340) was recorded after 0.5 and 5 minutes of incubation to monitor the rate of production of the HK catalytic product. HK activity was calculated based on changes in A340 readings and the standard curve of the reaction product.

To measure PFK activity, 30 μl of each sample was mixed with and incubated in 810 μl reaction Mix containing PFK substrate and coenzymes at 37 °C. Absorbance at 340 nm (A340) was recorded after 0.3 and 10 minutes of incubation to monitor the rate of production of the reaction production. PFK activity was calculated based on changes in A340 readings and the standard curve of the reaction product.

To measure CS activity, 10 μl of each sample was mixed with and incubated in 240 μl reaction Mix containing CS substrate and cofactors at 37 °C. Absorbance at 412 nm (A412) was recorded after 5 and 15 minutes of incubation to monitor the rate of production of the enzymatic reaction product. CS activity was calculated based on changes in A412 readings and the standard curve of the reaction product.

### Measurement of mitochondrial respiratory chain complex activities

Mitochondria were isolated as previously described^43^. Briefly, mouse lungs or human PBMCs were homogenized in isolation buffer (1 mM EGTA, 215 mM mannitol, 75 mM sucrose, 0.1% BSA, 20 mM HEPES, pH 7.2). Mitochondria were isolated by differential centrifugation, and homogenized. Mitochondrial respiratory chain complexes I and III enzyme activities were measured using respective assay kits (Nanjing Jiancheng Bio-engineering Institute, Nanjing, China). To measure complex I activity, 900 μl reaction Mix containing ubiquinone and reduced nicotinamide adenine dinucleotide (NADH) was incubated at 30 °C for 3 minutes before 100 μl sample was added. Absorbance at 340 nm (A340) was recorded immediately after the addition of sample and after further incubation for 3 minutes to monitor the rate of NAD production. Absorbance at 380 nm (A380) was also recorded at the two time points for correcting background absorbance. Total complex I activity was calculated based on rate of changes in the corrected A340 readings (A340-A380) and the NAD standard curve. A parallel reaction, in which a specific complex I inhibitor was added into the reaction Mix, was performed for each sample to determine non-specific complex I activity. Specific complex I activity for each sample was calculated by subtracting the non-specific activity from total complex I activity.

To measure complex III activity, 900 μl reaction Mix containing ubiquinol and 2-ferricytochrome c was incubated at room temperature in dark for 10 minutes before 100 μl sample was added. Absorbance at 550 nm (A550) was recorded immediately after the addition of sample and after further incubation for 5 minutes to monitor the rate of reduced cytochrome c production. Complex III activity was calculated based on changes in A550 readings and the reduced cytochrome c standard curve.

### Measurement of mitochondrial NAD, NAMPT, and NAD/NADH ratio

Mitochondria were isolated from mouse lungs, and homogenized. Mitochondrial nicotinamide adenine dinucleotide (NAD), NADH and nicotinamide phosphoribosyltransferase (NAMPT) were measured using corresponding assay kits (Nanjing Jiancheng Bio-engineering Institute, Nanjing, China). For the NAD assay, NAD/NADH was extracted from mitochondrial protein in extraction buffer. Each sample and NADH standard were treated with alcohol dehydrogenase to convert all NAD to NADH. The treated samples and NADH standard were then incubated in detection buffer containing MTT [3-(4, 5-Dimethylthiazol-2-yl)-2, 5-diphenyltetrazolium bromide] at room temperature for 1 hours. Absorbance at 570 nm (A570) was recorded to monitor the conversion of MTT to formazan. Total NAD/NADH was calculated based on change in A570 readings and the NADH standard curve. To measure NADH, sample was heated to 60 °C for 30 minutes followed by cooling down on ice before treatment with dehydrogenase. This heat treatment decomposes all NAD, but keeps NADH intact. Sample NADH level was then quantified as described above. Sample level of NAD was calculated by subtracting NADH from total NAD/NADH. NAD/NADH ratio was determined by dividing NAD by NADH.

NAMPT was quantified using a competitive immunoassay. Fifty μl of each sample (or NAMPT standard) and 50 μl working solution containing biotin-conjugated antigen were added into a plate well coated with NAMPT-capture antibody, and incubated at 37 °C for 1 hour. Following washing, 100 μl streptavidin-conjugated horseradish peroxidase (HRP) was added, and incubated at 37 °C for 1 hour. Plate was washed and 100 μl HRP substrate added. After incubation at 37 °C for 10 minutes, reaction was stopped, and absorbance at 450 nm (A450) recorded. Mitochondrial NAMPT level was calculated based on A450 readings and the NAMPT standard curve.

### Determination of mitochondrial ROS generation, and mitochondrial antioxidant enzyme activities

Mitochondrial ROS production was monitored using 7′-dichlorofluorescein diacetate (DCFH-DA) fluorescence probe (Beyotime Company, Guangzhou, China). Briefly, isolated mitochondria (0.5 mg/ml) were incubated with 10 μM DCFH-DA at 37 °C for 1 hour, and the fluorescence intensity was measured using microplate fluorometer (Perkin-Elmer, Waltham, USA) at excitation of 488 and emission of 525 nm.

Activities of mitochondrial Mn-superoxide dismutase (Mn-SOD) and glutathione peroxidase (GSH-Px) activities were determined using Mn-SOD and GSH-Px assay kits (Nanjing Jiancheng Bio-engineering Institute, Nanjing, China). The Mn-SOD assay kit measures superoxide anion oxidation of a chromagen into an oxidative product that absorbs light. SOD activity is determined as inhibition of the chromagen oxidation. Samples and saline were treated with an inhibitor that inactivates Mn-SOD, but has no effect GuZn-SOD activity. The treated samples and saline were mixed with reaction/detection solution in GuZn-SOD-test and GuZn-SOD-control wells, incubated at 37 °C for 40 minutes, and absorbance at 550 nm (A550) recorded. Saline and untreated samples were used for measuring total SOD activity. Total and GuZn-SOD activities were determined based on changes in A550 readings. Mn-SOD activity was calculated by subtracting GuZn-SOD from total SOD activity.

GSH-Px activity was assayed based on the reaction of reducing free hydrogen peroxide to water. In this reaction, GPx converts reduced glutathione (GSH) to oxidized glutathione (GSSG). GSH-Px assay kit measures GSH-Px activity based on GSH consumption. Each sample (200 μl) was mixed with and incubated in GSH solution at 37 °C for 5 minutes, and then in reaction buffer for further 5 minutes. Following addition of application buffer, the reaction was centrifuged, and supernatant collected. Supernatant and GSH standard were incubated in developer solution at room temperature for 15 minutes. Absorbance at 412 nm (A412) was recorded every 2 minutes for 10 minutes. GSH-Px activity was calculated based on changes in A412 readings and the GSH standard curve.

### Histology and transmission electron microscopy

Mouse lungs were fixed in 10% neutral buffered formalin and processed. Paraffin-embedded sections (4 μm) were prepared and stained with hematoxylin and eosin. For electron microscopy, mouse lungs were rapidly excised and fixed with 2% paraformaldehyde and 2.5% glutaraldehyde in 0.1 M sodium cacodylate buffer and postfixed with 1% osmium tetroxide followed by 1% uranyl acetate. Fixed lungs were dehydrated through a graded series of ethanol and embedded in LX112 resin (LADD Research Industries, Williston, VT, USA). Sections (80 nm) were cut on a Reichert Ultracut UCT, stained with uranyl acetate followed by lead citrate, and viewed on a JEOL 1200EX transmission electron microscope.

### Measurements of BALF IgE, cytokine and reactive oxygen species (ROS)

BALF was centrifuged at 7000 g for 4 minutes to remove debris. BALF levels of total and OVA-specific IgE were measured using mouse IgE ELISA kit (Bethyl Laboratories, Montgomery, AL, USA) and Legend MAX mouse OVA-specific IgE ELISA Kit (Biolegend, San Diego, CA, USA). BALF IL-4 and IL-5 were measured using mouse IL-4 and IL-5 ELISA kits (R&D Systems, Minneapolis, MN, USA).

BALF leukocyte ROS production was measured using membrane-permeable fluorescent probe, DCFH-DA (Beyotime Company, Guangzhou, China). One ml of BALF containing 1 × 10^5^ leukocytes was mixed and incubated with 20 μM DCFH-DA at 37 °C for 20 minutes. Fluorescence intensity of the oxidized DCFH-DA was read using microplate fluorometer (Perkin-Elmer, Waltham, USA) at excitation of 488 and emission of 525 nm.

### BALF cell count and differential cell count

BALF was centrifuged. The pellet was resuspended in PBS. Total cell number was counted with haemocytometer after cell viability determination by trypan blue exclusion test. For differential cell counts, cells were centrifuged onto cytospin slides, differentially stained with Diff-Quik stain reagent (Beyotime Institute of Biotechnology, Nanjing, China) and counted.

### Measurement of airway responsiveness to methacholine

Airway responsiveness was assessed 24 hours after final OVA challenge. Mice were anesthetized (nembutal 60 mg/kg, i.p.), trachea cannulated, mechanically ventilated and placed in a plethysmograph chamber with a pneumotachograph connected to an AniRes2005 pulmonary mechanics data collection and analysis system (BestLab, Beijing, China). This system records lung volume, flow and pressure changes during expiration or inspiration, and analyzes airway responsiveness based on change in airway resistance. Mice were equilibrated in the chamber for 20 minutes, and then sham-challenged with PBS or challenged with 3 concentrations of nebulized methacholine (3, 9, and 27 mg/ml) for 1 minute with a 20 minute interval. Airway responsiveness was recorded for 3 minutes before and after each challenge. Mean of the 3 minute recordings was reported as airway responsiveness.

### Western blot

Total, cytoplasmic or nuclear protein was extracted from mouse lungs. Equal amounts of protein (30 μg) were separated on 10% SDS-PAGE gel, and transferred to polyvinylidene fluoride membrane (Millipore, Billerica, MA, USA). The membrane was cut horizontally into stripes corresponding to different molecular weight ranges, based on protein size markers. Stripes that are expected to contain hypoxia inducible factor (HIF)-1α, peroxisome proliferator-activated receptor-γ coactivator (PGC)-1α, sirtuin 1, sirtuin 3, sirtuin 6, β-actin, lamin B or NF-κB p65 protein were used for immunoblotting to detect each specific protein band. Stripes were blocked with 5% non-fat dry milk in TBST (10 mM Tris-HCl pH 7.5, 150 mM NaCl, 0.1% Tween-20), incubated overnight at 4 °C with antibodies specific to HIF-1α, PGC-1α, sirtuin 1, sirtuin 3, sirtuin 6, β-actin, lamin B or NF-κB p65 (Bioss Inc, Beijing, China or Cell Signaling Technology, Danvers, MA, USA). Stripes were washed, incubated with horseradish peroxidase-conjugated secondary antibody (Bioss Inc., Beijing, China), and washed. Immunoreactive bands were visualized using ECL reagents (Thermo Scientific, Waltham, MA, USA). This modified western blot protocol improved sensitivity and specificity, and reduced the quantity of antibody usage. However, cutting the whole membrane into stripes has eliminated the possibility of obtaining full-length image of the western blot. Acetylated HIF-1α, acetylated PGC-1α and acetylated p65 were analyzed by immunoprecipitation of HIF-1α, PGC-1α and NF-κB p65 proteins followed by western blot using acetylated-lysine antibody (Cell Signaling Technology, Danvers, MA, USA).

### Real-Time PCR

RNA was isolated from mouse lungs using the RNeasy mini kit (Qaigene, Germantown, MD, USA), and reverse transcribed into cDNAs using Sensiscript RT kit (Promega, Madison, WI, USA). Real-time PCR was performed using an ABI Prism 7900 sequence detection system (Applied Biosystems, Foster city, CA, USA) with SYBR Green PCR master mix following standard protocol. The primer sequences were given in Table 2. Results were analyzed using the 2^−ΔCt^ method. Each sirtuin mRNA expression was normalized to its corresponding GADPH mRNA.

**Table 2:** Primers Used for Real Time PCR.

Gene	Forward Primer	Reverse Primer
GAPDH	5′-AGGTCGGTGTGAACGGATTTG-3′	5′-GGGGTCGTTGATGGCAACA-3′
Sirtuin 1	5′-ATGACGCTGTGGCAGATTGTT-3′	5′-CCGCAAGGCGAGCATAGAT-3′
Sirtuin 2	5′-GCGGGTATCCCTGACTTCC-3′	5′-CGTGTCTATGTTCTGCGTGTAG-3′
Sirtuin 3	5′-GAGCGGCCTCTACAGCAAC-3′	5′-GGAAGTAGTGAGTGACATTGGG-3′
Sirtuin 4	5′-GATTGACTTTCAGGCCGACAA-3′	5′-GCGGCACAAATAACCCCGA-3′
Sirtuin 5	5′-AATATGGCAGACTTTCGGAAGTG-3′	5′-ACACCTGTGATGGGTTTCGAG-3′
Sirtuin 6	5′-CTCCAGCGTGGTTTTCCACA-3′	5′-GCCCATGCGTTCTAGCTGA-3′
Sirtuin 7	5′-GCACTTGGTTGTCTACACGG-3′	5′-TGTCCATACTCCATTAGGACCC-3′

### Statistical analysis

Data were expressed as means ± SEM. Unpaired t test was used to compare the means of two independent groups. For multiple group comparison, data was analyzed using one-way ANOVA followed by *post hoc* analysis using Holm-Sidak method. A p-value less than 0.05 was considered significant.

## Supplementary information

Supplementary Information.
