# Supplementary material for: Hydrogen Attenuates Allergic Inflammation by Reversing Energy Metabolic Pathway Switch
Source: Sci Rep. 2020 Feb 6;10:1962. doi: 10.1038/s41598-020-58999-0 (PMC7005324; doi:10.1038/s41598-020-58999-0)
Supplement: Supplementary file 1 — Supplementary Information. [file 41598_2020_58999_MOESM1_ESM.pdf]

Hydrogen Attenuates Allergic Inflammation by Reversing Energy Metabolic Pathway Switch

Yinghao Niu [a], Qingrong Nie [b], Liping Dong [a], Jihua Zhang [a], Shu Fang Liu [a], Wei Song [c], Xiaopei Wang [a], Guangli Wu [a] and Dongmei Song [a]

[a] Departments of Otolaryngology and Clinical Biobank, the First Hospital of Hebei Medical University, Shijiazhuang, 050031, Hebei, China.

[b] Department of Respiratory and Critical Care Medicine, Liangxiang Hospital of Beijing Fangshan District, Fangshan, Beijing, 102401, China.

[c] Department of Psychiatry and Behavioural Neurosciences, Faculty of Health Sciences, McMaster University, ON L8N 3Z5, Canada.

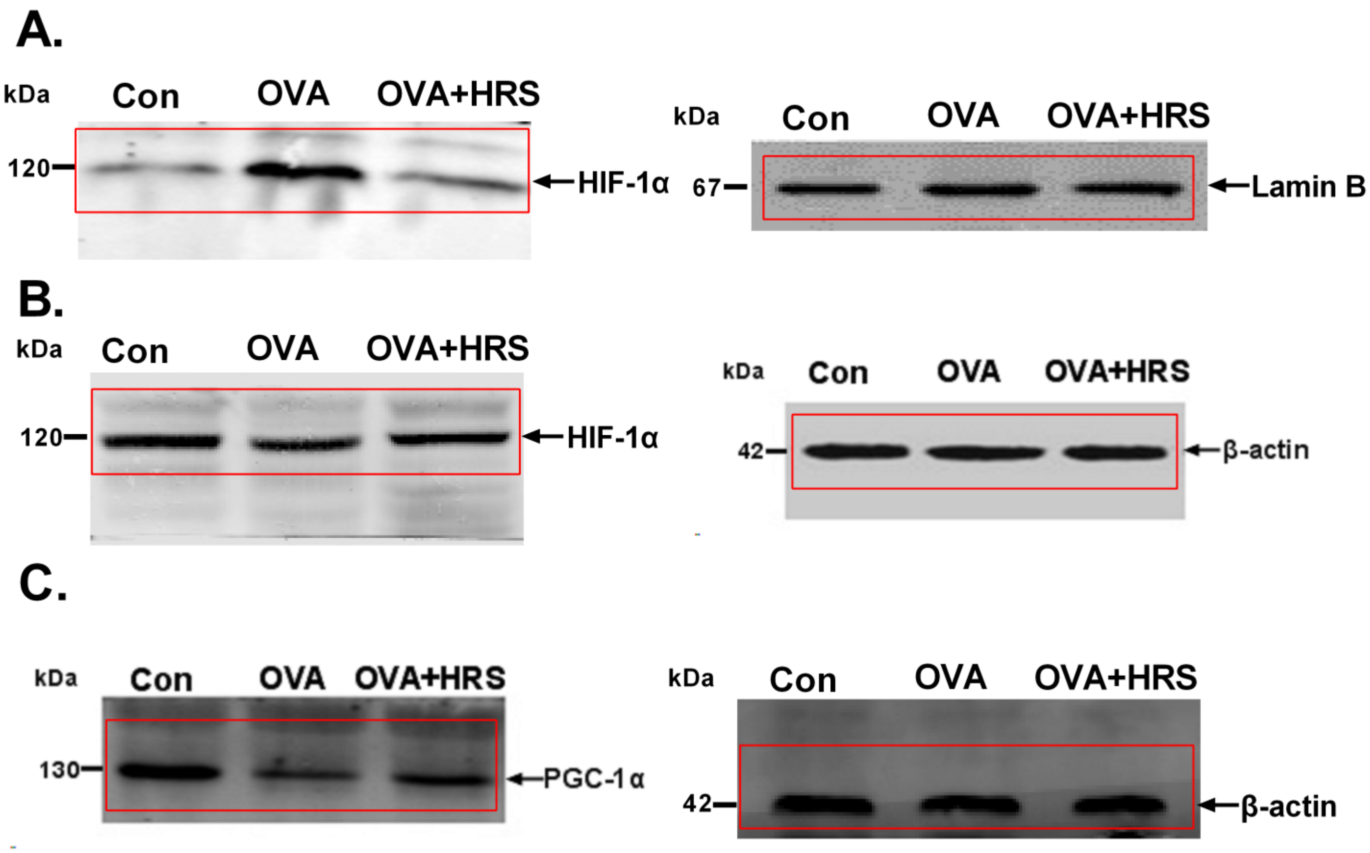

Supplementary Figure 1. Original blots for Western blot images presented in main figures 3A, 3B and 3C.

Equal amounts of protein were separated on SDS-PAGE gel and transferred to polyvinylidene fluoride membrane. The membrane was cut horizontally into stripes corresponding to different molecular weight ranges, based on protein size markers. Stripes that are expected to contain HIF-1α, lamin B, PGC-1α or β-actin protein were used for immunoblotting to detect HIF-1α (**A** and **B,** left), lamin B (**A**, right), PGC-1α (**C**, left) or β-actin band (**B** and **C**, right). This modified western blot protocol improved sensitivity and specificity, and reduced the quantity of antibody usage. However, cutting the whole membrane into stripes has eliminated the possibility of obtaining full-length image of the western blot. The red lines outline the cropped images presented in main figures 3A, 3B and 3C.

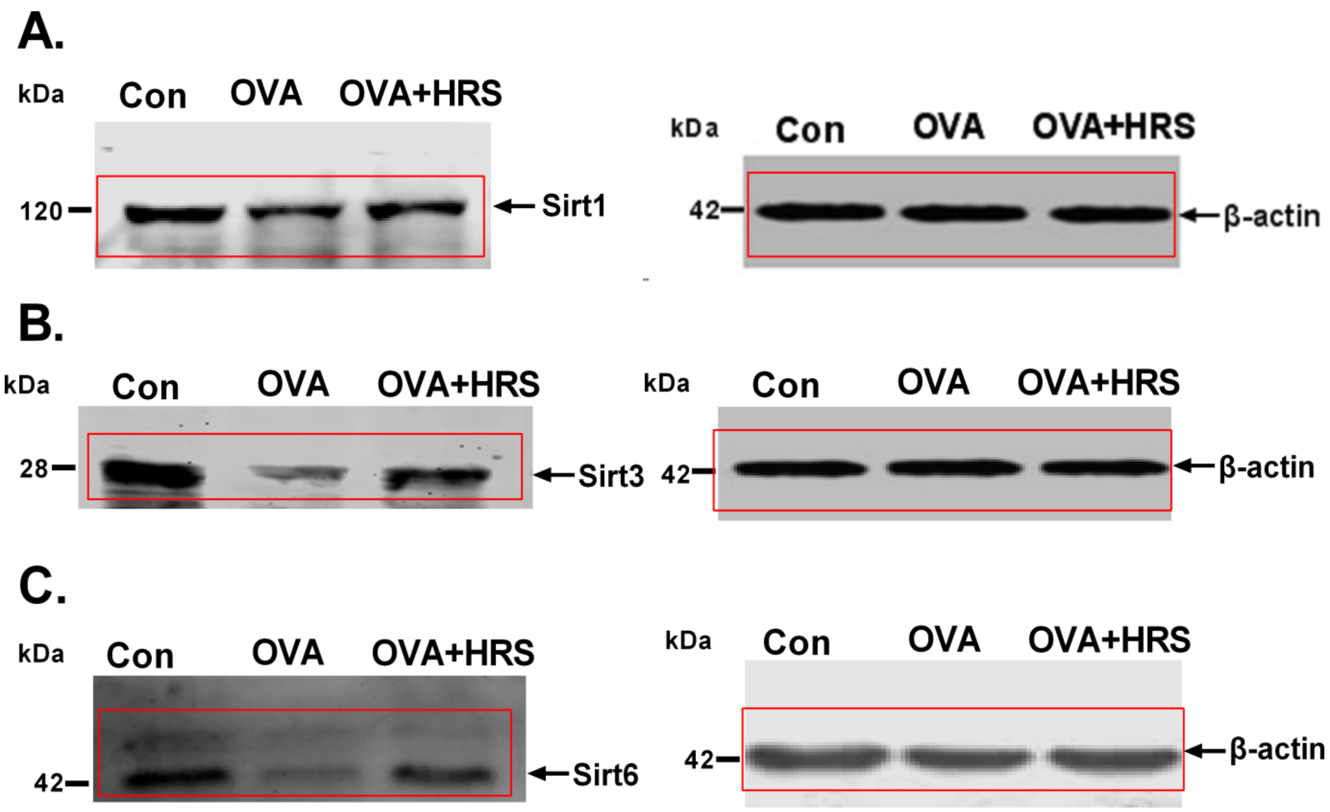

Supplementary Figure 2. Original blots for Western blot images presented in main figures 4C, 4D and 4E.

Equal amounts of protein were separated on SDS-PAGE gel and transferred to polyvinylidene fluoride membrane. The membrane was cut horizontally into stripes corresponding to different molecular weight ranges, based on protein size markers. Stripes that are expected to contain sirtuin (Sirt) 1, 3 or 6, or β-actin protein were used for immunoblotting to detect Sirt 1, 3 or 6 (**A,** **B** or **C,** left) or β-actin band (**A**, **B** and **C**, right). This modified western blot protocol improved sensitivity and specificity, and reduced the quantity of antibody usage. However, cutting the whole membrane into stripes has eliminated the possibility of obtaining full-length image of the western blot. The red lines outline the cropped images presented in main figures 4C, 4D and 4E.

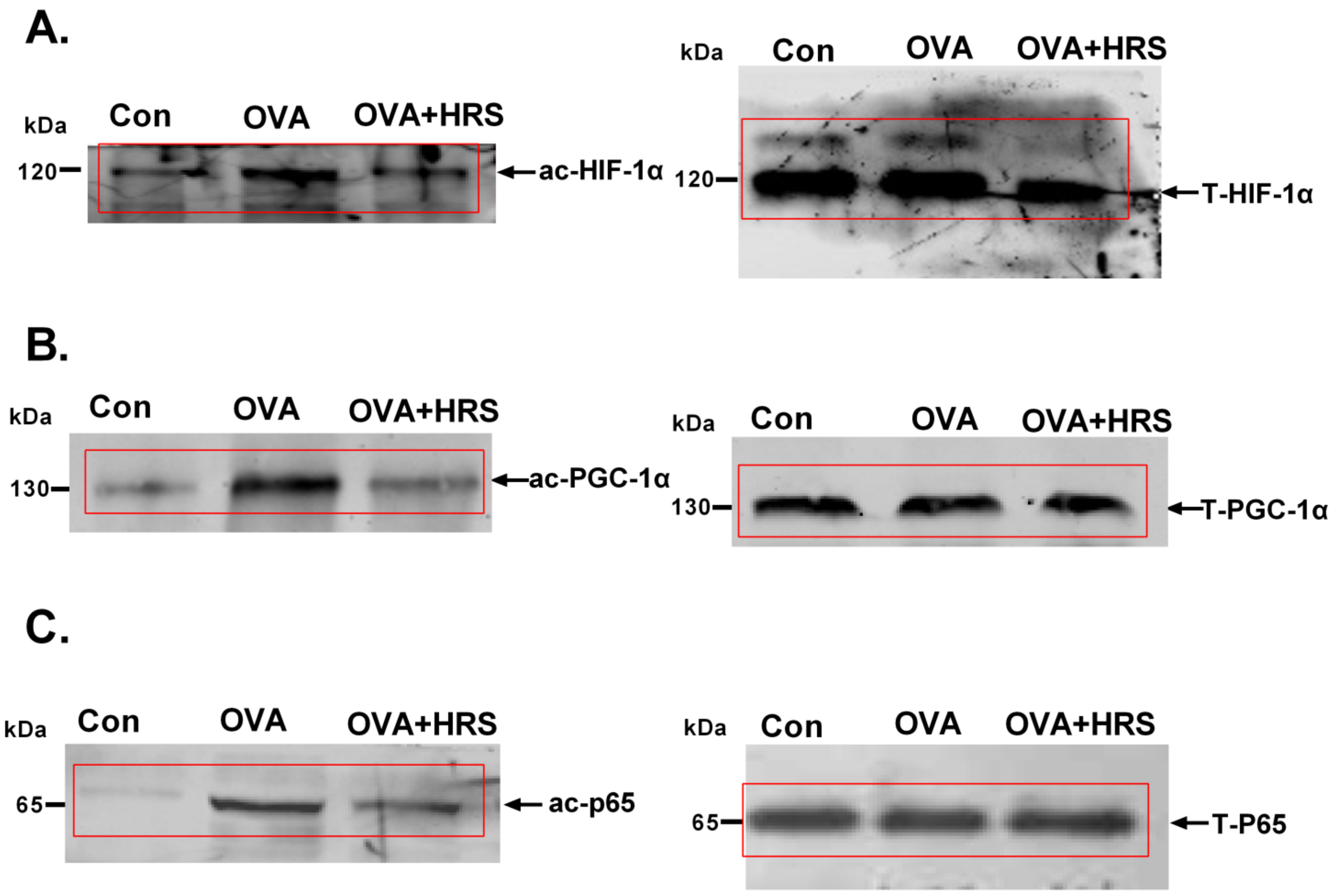

Supplementary Figure 3. Original blots for Western blot images presented in main figures 5A, 5B and 5C.

Protein was separated on SDS-PAGE gel and transferred to polyvinylidene fluoride membrane. The membrane was cut horizontally into stripes corresponding to different molecular weight ranges, based on protein size markers. Stripes that are expected to contain HIF-1α, PGC-1α or NF-κB p65 protein were used for immunoblotting to detect acetylated and total HIF-1α, (**A**), acetylated and total PGC-1α (**B**) or acetylated and total p65 (**C**). This modified western blot protocol improved sensitivity and specificity, and reduced the quantity of antibody usage. However, cutting the whole membrane into stripes has eliminated the possibility of obtaining full-length image of the western blot. The red lines outline the cropped images presented in main figures 5A, 5B and 5C.
